# Carbonyl Reductase 3 (CBR3) Mediates *9-cis-Retinoic Acid*-Induced Cytostatis and is a Potential Prognostic Marker for Oral Malignancy

**DOI:** 10.2174/1874210600802010078

**Published:** 2008-06-09

**Authors:** Shuri Ohkura-Hada, Nobuo Kondoh, Akiyuki Hada, Masaaki Arai, Yutaka Yamazaki, Masanobu Shindoh, Yoshimasa Kitagawa, Masayuki Takahashi, Toshifumi Ando, Yasunori Sato, Mikio Yamamoto

**Affiliations:** 1 Department of Biochemistry, National Defense Medical College, 3-2 Namiki, Tokorozawa-shi; 2 Department of Oral surgery, National Defense Medical College, 3-2 Namiki, Tokorozawa-shi; 3 Department of Oral Diagnosis, Hokkaido University Graduate School of Dental Medicine, Sapporo-shi; 4 Department of Oral Pathology and Diagnosis, Hokkaido University Graduate School of Dental Medicine, Sapporo-shi; 5 Department of Biochemistry, Asahi University School of Dentistry, 1851 Hozumi, Mizuho-shi, Japan

## Abstract

The molecular mechanisms of growth suppression by retinoic acid (RA) were examined. Our results suggest that the cytostatic effects of RA could be mediated by the activation of endogenous CBR3 gene in oral squamous cell carcinomas (OSCCs), and the expression is a potential marker for oral malignancy.

## INTRODUCTION

The retinoic acids are potent modulators of cell growth, differentiation and apoptosis in a variety of tissues [1]. Animal and *in vitro* studies, as well as clinical trials, clearly demonstrate a role for retinoids as cytostatic agents in the prevention of epithelial carcinogenesis [2, 3]. Retinoids suppress the growth and the aberrant squamous phenotype of head and neck squamous cell carcinomas, at least in part, *via *the regulation of receptor complexes [4]. There are several retinoid analogues that are widely used for therapeutic purposes, including all-trans retinoic acid (ATRA), *9-cis-*RA and *13-cis-*RA. Among them, *9-cis-*RA is reportedly a more potent agent than ATLA or *13-cis-*RAfor growth suppression and the induction of apoptosis in acute myeloblastic leukemia cells [5]. *9-cis-*RA also inhibits the cell growth of neuroblastoma [6], breast [7], pancreas carcinomas [8] as well as osteosarcoma [9] and skin cancers [10]. In addition, *9-cis-*RA suppresses cell migration and reduces the synthesis of extracellular matrix of vascular smooth muscle cells [11]. However, the mechanism of these cytostatic effects is not well understood.

In this study, we investigated the effects of retinoic acid on the expressions of a wide variety of genes, and evaluated it as a mediator for the suppression of transformed phenotypes for oral squamous cell carcinoma (OSCC) cells.

## MATERIALS AND METHODS

### Tissue Specimens and OSCC Cell Lines

Human OSCC cell line Ca9-22 was provided by the Japanese Cancer Research Resources Bank (JCRB). Cells were maintained in Dulbecco’s modified Eagle’s minimum essential medium (DMEM) supplemented with 10% fetal bovine serum (FBS). Culture media was changed on alternate days during the experiments.

In addition to OSCCs, hyperplastic and mildly, moderately and severely dysplastic tissues were included in this study. These tissue samples had been surgically resected in the Dental Hospital of Hokkaido University from February 1998 to April 2004. Each sample was macrodissected, and the stromal tissues were carefully removed as well as possible by a pathologist, then stored at -100 ^o^C. Clinico-pathological features of OSCCs used in two sets of experiments are listed in Tables **2** and **3**, respectively. All procedures were undertaken following informed consent from each patient, and adhered to the ethical guidelines of the Dental Hospital of the Hokkaido University School of Dentistry (Sapporo, Japan).

### cDNA Microarray Analysis

cDNA microarray analysis was performed using IntelliGene HS Human expression chips containing ~ 16,600 probe sets (Takara). Briefly, 4 µg of total RNA was used for double-stranded cDNA probe synthesis with a T7 oligo (dT) primer. Each cDNA fragment was subjected to RT amplification that incorporated aminoalyl-UTP (Ambion), which was coupled with either Cy3 or Cy5 (Amersham Biosciences). After purification through a Microcon 30 spin column (Millipore), the amplified RNAs were combined and hybridized to the IntelliGene HS Human expression chips in hybridization buffer containing Cot-1 DNA (Invitrogen). Each slide contained duplicate sets of samples and Cy3 /Cy5 labeling of the cDNA probes was reversed in duplicate experiments. After overnight hybridization at 70 (C, the slides were washed, scanned for Cy3 and Cy5 fluorescence using a ScanArray Express microarray reader (Perkin-Elmer) and the signal was quantified using ScanArray Express software (Perkin-Elmer).

### Quantitative RT-PCR Analysis

To validate the microarray analysis, we performed Quantitative RT-PCR [11] in the cell lines and the tissue samples. Total RNA isolates (2μg) were reverse-transcribed using oligo (dT)12-18 primers and Superscript II (Invitrogen). Oligo primers for RT-PCR were designed by Primer Express software (Applied Biosystems) and are listed in Table **1**. Each PCR amplification was carried out in triplicate in an ABI Prism 5700 Sequence Detection System using Sybr Green Mastermix (Applied Biosystems) for 15 min at 95 (C for the initial denaturing, followed by 40 cycles of 95 (C for 30 sec and 60 (C for 1 min*.* The expression values for each gene were normalized to the expression levels of the S5 ribosomal protein transcript (Genbank accession No, NM 001009 ; primer pairs, 5’- GAG CGC CTC ACT AAC TCC ATG ATG A –3’ and 5’- CAC TGT TGA TGA TGG CGT TCA CCA –3’), which was used as an internal control. Statistical analysis was performed using Welche’s *t*-test.

### Plasmid Construction

A 0.87-kb cDNA insert corresponding to 214-1083 and encoding CBR3 ORF (GenBank accession No.; NM 001236) was generated by RT-PCR, as previously described [10]. The primers used for amplification were 5’- GCT CCC CGC TCA GCC ATG TCG T –3’ and 5’- AGC AAG CTC CGA AGC AGA CGT TTA CCA G–3’. The amplified cDNAs were cloned into a pcDNA4/TO vector (Invitrogen) and sequencing examination was performed.

### Transfection Experiments

CA9-22 cells were transfected with 5 μg of the *CBR3* expression vector using Superfect reagent (Qiagen) and selected in medium containing zeocin (0.1 mg/ml; Invitrogen).

### Protein Extraction and Immunoblotting

Protein extraction and ECL western blotting analysis (Amersham Pharmacia) were performed as described previously [7] using anti-CBR3 antibody (ab21958, Abcam).

### Wound-Healing Assay

The migration potential of cells was determined by a wound-healing assay. Cells were grown on fibronectin-coated culture dishes to confluence. A wound was made in the middle of the culture dish using pipet chips. Cells were photo-micrographed at the indicated times.

### Statistical Analysis

Statistical significance of differential *CBR3* mRNA expression between OSCCs and pre-cancerous tissues was evaluated by Welche’s t-test. The expression levels of *CBR3* mRNA among 64 OSCC tissues of several clinico-pathological appearances were evaluated by Mann Whitney’s U-test or chi-square test.

## RESULTS

### Growth Inhibition by *9-cis-Retinoic Acid* (RA)

**Fig. (1).**
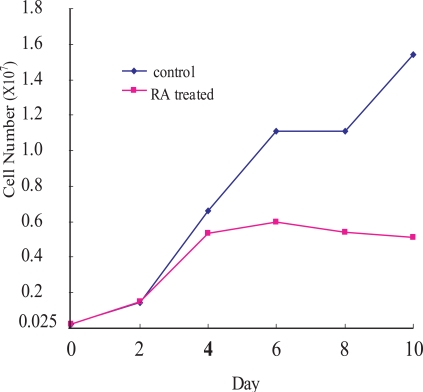
Cell growth of Ca9-22 cells. RA-treated (red line) and untreated (blue line) Ca9-22 cells. Ca9-22 cells were treated with 10-^6^M of *9-cis *RA. *Ordinate*: cell number; *abscissa*: days after cells were seeded, with or without *9-cis* RA. The experiments were repeated twice, and a representative result is shown.

To examine the growth inhibitory effect of RA on OCSS cells, Ca9-22 cells were treated with 10^-6 ^M *9-cis-*RA for different periods of time (red line in Fig. **1**). Ca9-2 cells were growing until day 2 after the RA treatment, after which the cell growth rate declined, and was significantly suppressed after day 4 until day 10. By comparison, untreated control cells showed continuous growth during the experiment.

### Differentially Expressed Genesfor RA-Treated and Untreated Ca9-22 Cells

Because cell growth did not change until day 2, while it began to decline at day 4 after the RA treatment, we speculated that RA-regulated growth-associated genes may have been recruited during day 3 after the RA treatment. Thus, in order to identify differentially expressed genes comparing RA-treated and untreated Ca9-22 cells, cDNA microarray analyses were performed using RNA samples extracted from the cells that were maintained in the presence or absence of RA for 3 days. Among 16,600 target cDNAs, we focused upon differentially expressed genes with greater than 2-fold changes in their expressions.

We tentatively identified 25 up- and 33 down-regulated gene candidates due to RA treatment. These marker gene candidates were further examined by RT-PCR analysis. Ultimately, we focused on 14 up-regulated and 17 down-regulated genes that showed significant differences between RA-treated and untreated cells (Table **1**). Based upon time course analysis of the expression patterns of the 14 up-regulated genes in RA-treated cells, we tentatively classified them into three types.

**Fig. (2).**
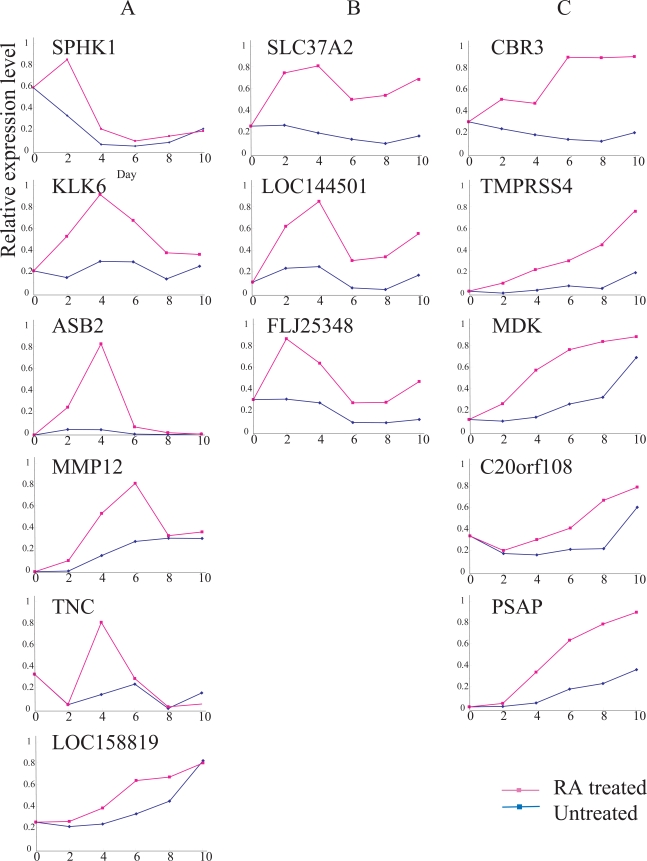
Expressions of 14 RA-up-regulated genes. *Ordinate*, relative expression level; *abscissa*, days after Ca9-22 cells were seeded with (red line) or without (blue line) 9-cis RA. See Results for explanations of gene types ‘A’, ‘B’ and ‘C.’ The expression of each gene was examined, at least, twice; representative results are demonstrated.

A type genes were transiently up-regulated within a few days after the RA treatment, and down-regulated thereafter even in the presence of RA. C types showed gradual, but continuous up-regulation in the presence of RA., B types showed intermediate expressions between types A and C (Fig. **2**). Some of the genes whose expressions were up-regulated by RA included *SPHK1, LOC158819, MDK, C20orf08, PSAP, MMP12, TNC *and* LOC144501*. *FLJ25348* expression fluctuated due to cell growth conditions, even in the absence of RA. In contrast, the expressions of *KLK6, ASB, SLC37A2, CBR3 *and* TMPRSS4* were relatively unchanged during the same period of time (Fig. **2**).

**Fig. (3).**
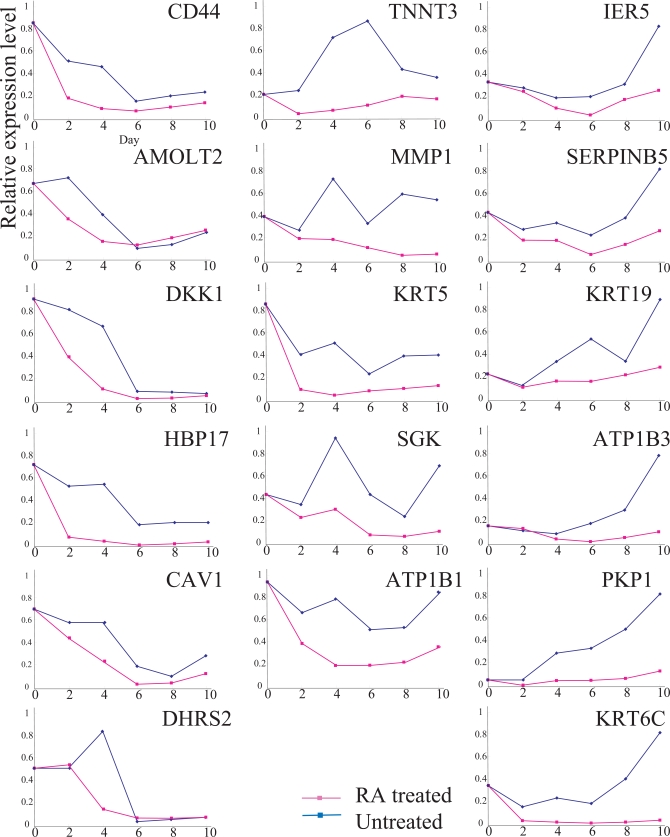
Expressions of 17 RA-down-regulated genes. Explanatory notes as in Fig. (**2**). The expression of each gene was examined, at least, twice; representative results are demonstrated

We also compared the expressions of 17 RA down-regulated genes for RA-treated and untreated Ca9-22 cells (Fig. **3**). Time course analysis demonstrated that the expressions of the 17 genes were reduced in RA-treated cells compared to that in untreated cells. However, the expressions of all 17 genes fluctuated significantly, even in the absence of RA, suggesting that their expressions were influenced by the cell growth conditions rather than RA growth regulation. Therefore, we focused on the *SLC37A2* and *CBR3* genes.

The expression of *CBR3 *was significantly up-regulated by day 2, and elevated expression continued until day 6. In contrast, the expression of *SLC37A2* was also elevated by day 2, and maximally elevated by day 4. The expressions of both genes were relatively unchanged in the absence of RA (Fig. **2**).

### Expression of CBR3 in OSCC and Pre-Cancerous Lesions

**Fig. (4).**
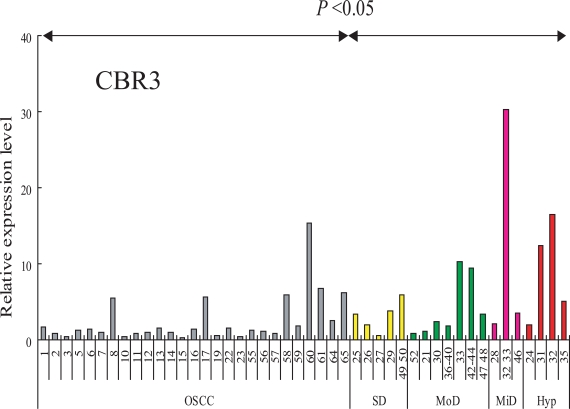
Expression of CBR3 mRNA in cells and tissues. CBR3 mRNA was determined by QRT-PCR. *Ordinate*, relative expression level: *abscissa*, samples from OSCC (gray bars), severe dysplasia (yellow bars), moderate dysplasia (green bars), mild dysplasia (pink bars) and hyperplasia (red bar).

RT-PCR analysis was performed in order to analyze the transcript levels of the *CBR3* gene in our sample set of 27 OSCCs (clinico-pathological features are listed in Table **2**), and 19 leukoplakia (LP) tissues that included 4 hyperplasias and 3 mild, 7 moderate and 4 severe dysplasias (Fig. **4**). The expression of *CBR3 *was significantly higher in LP tissues than in OSCCs (P< 0.05). We also examined the expression of *SLC37A2 *mRNA in these LP and OSCC tissues, although no significant differences between OSCCs and LPs were observed (data not shown).

We further examined whether the expression of *CBR3* mRNA was associated with the clinico-pathological features of 64 OSCC samples (Table **3**). Interestingly, *CBR3 *mRNA expression was significantly down-regulated in highly invasive OSCCs (>YK-3) than in less invasive ones (p<0.018). *CBR3 *expression was not affected by age, gender, T classification or metastatic potential (Table **4**). Our results demonstrate that the expression of *CBR3* mRNA was significantly reduced in OSCCs, especially in highly invasive cancers compared with pre-malignant dysplasias and hyperplasias.

### Functional Significance of CBR3 Expression in OSCC Cells

Our results, using both cell lines and tissues, demonstrated that the reduction of *CBR3 *expression was closely associated with growth suppression by RA. It was also associated with malignant transformation of oral epithelia toward invasive OSCCs. To assess the functional significance of CBR3 in OSCCs, we introduced the CBR3 expression vector into Ca9-22 cells. Three sub-clones, pcDNA1 CBR3-12, -14 and –16, were isolated. As shown in Fig. (**5**), the expression of CBR3 protein in pcDNA1 CBR3-16 cells was similar to that in RA-treated Ca9-22 cells, while in pcDNA1 CBR3-12 and -14 it was higher. The expression in control cells was undetectable.

**Fig (7):**
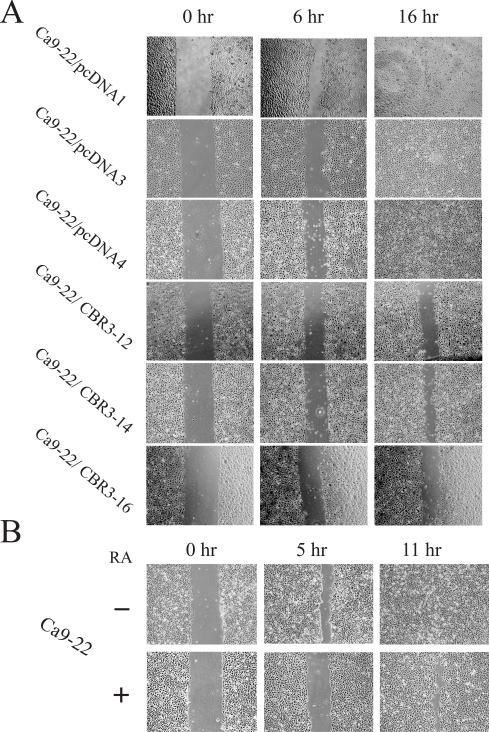
Cell mobility assay. Results for (A) CBR3-transfected and vector transfected or (B) *9-cis* RA-treated and untreated Ca9-22 cells.

As shown in Fig. (**6**), the cell growth rate was significantly reduced in the 3 *CBR3 *transfectants compared with 3 independently isolated vector-transfected cells. We next examined the effect of CBR3 and RA on the migratory potential of OSCC cells using a wound healing assay. As shown in Fig. (**7**), pcDNA1 CBR3-12, -14 and -16 cells had lower migration potentials compared with vector-transfected control cells. In addition, we confirmed that the migration potential was reduced in RA-treated parental Ca9-22 cells compared with untreated cells (Fig. **7**). We also examined the effect of CBR3 on the invasive potential of OSCC cells by using an *in vitro *matrigel assay, although no significant differences were observed between *CBR3*-transfected and –untransfected Ca9-22 cells (data not shown).

Our results demonstrate that the expression of CBR3 protein causes reduced cell growth and migration potential in pre-cancerous epithelium or in OSCC cells. Our results sug-gest that the cytostatic effects of RA on OSCC cells could be mediated, at least in part, by the up-regulation of the CBR3 product. Our results also suggest that down-regulation of CBR3 expression could be a prerequisite for pre-malignant to malignant transition of oral epithelium, and progression toward invasive OSCC.

## DISCUSSION

The anti-cancer activities of retinoic acids and their derivatives are attributable to three actions: cell-differentiation, growth inhibition and apoptosis [4]. Among these, RA-dependent growth suppression is associated with inhibition of MAPK pathways, and up-regulation of PKC isoforms and cyclin dependent kinases *via *up-regulation of p21^WAF1/CIP1^ and p27^kip1^ [4, 12, 13]. RA also exerts an anti-proliferative effect on tumors through up-regulation of interferon regulatory factor 1 (IRF1) and downstream INF-stimulated genes [4]. An INF-stimulated gene, XIAP-associated factor 1 (XAF1), which was induced by RA in colon cancers resulted in growth suppression [14]. A member of the high mobility group (HMG) box gene family transcription factor, SOX9, is also stimulated by RA, and involved in the RA-mediated growth suppression in breast cancer cells [15].

In this report, we first demonstrated that *9-cis-*RA induced *CBR3* expression in an OSCC cell line, Ca9-22. The induced expression of the CBR3 protein in Ca9-22 cells caused suppression of proliferation, suggesting that CBR3 is also a mediator of cytostatic effects by RA. Nuclear retinoic acid receptors are ligand-dependent transcription factors that control activity *via *binding to retinoic acid responsive elements (RAREs) [4]. We observed a potential RXR responsive element, which could be a target for the *9-cis-*RA-receptor complex, that was located on the upstream region (-1924) of the transcriptional start site for the CBR3 gene (Confirmed by a TESS-string search, TessMaster@cbil upenn.edu; data not shown). However, maximal up-regula-tion of *CBR3*mRNA by RA required a considerable period of time (longer than 6 days; Fig. **2**). This suggests that, for this gene’s activation, there may be indirect mechanisms rather than direct transactivation *via *RARE.

We also demonstrated that the *CBR3* mRNA expression was markedly reduced in OSCCs compared to that in pre-malignant dysplasias and hyperplasias. Among OSCCs, the expression of *CBR3* was significantly down-regulated in highly invasive tumors compared with less invasive ones. This was corroborated by our observation that enforced expression of the CBR3 protein in Ca9-22 cells suppressed cell mobility.

Our results are also in accord with the following observations. CBR expression was markedly suppressed in metastatic ovarian cancers, and modulated metastatic potential [16]. CBR expression was a good prognostic factor in non-small-cell lung cancer [17]. The gene also plays important roles for the reduction of metastatic behavior of mouse tumor cells [18]. We also examined the effect of CBR3 on the invasive potential, however no significant differences were observed between *CBR3*-transfected and –untransfected Ca9-22 cells (data not shown). Since, several OSCC cell lines, including Ca9-22 cells did not show tumorigenicity in immune-defective nude mice (our unpublished data), the effects of exogenous *CBR3* gene upon invasive potential may be under estimated in Ca9-22 cells.

CBR was originally identified as a cytosolic NADPH-dependent oxido-reductase that reduced a wide variety of carbonyl compounds [19]. It has been isolated from a number of human tissues including brain, liver, breast, ovary and placenta [20, 21, 22]. Its actions are identical to prostaglandin 9-ketoreductase that inactivates prostaglandin E2 (PGE2) by converting to PGF2a [23]. PGE2 is a potent stimulator of vascular endothelial growth factor (VEGF) [24], and proliferation and invasiveness of cancers [25], while RA inhibits PGE2 synthesis [26]. Thus, we compared PGE2 production in *CBR3*-transfected and un-transfected Ca9-22 cells, although no significant differences were observed (data not shown). Further analysis of the function and regulatory mechanism of the *CBR3* gene should clarify its roles in oral malignancy.

## Figures and Tables

**Fig. (5).**
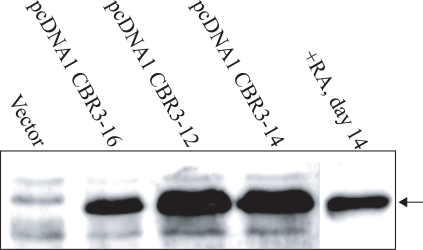
Expression of CBR3 protein in Ca9-22 cells treated with RA or transfected with *CBR3* expression vectors.

**Fig (6):**
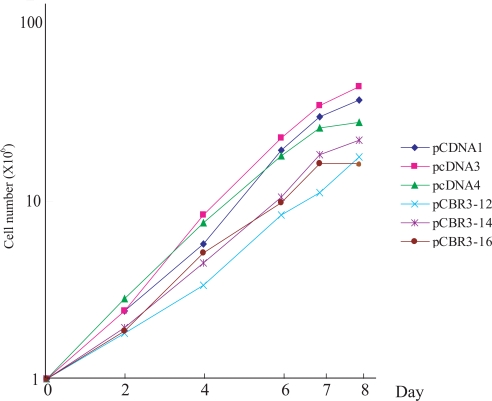
Cell growth of *CBR3*-transfected and vector-transfected Ca9-22 cells. *Ordinate*, cell number; *abscissa*, days after cells were seeded. The experiments were repeated twice, and a representative result is shown.

**Table 1.** Effects of 9-cis-RA on the Gene Expression in Ca9-22 Cells.

Up-Regulated Genes	GenBank No.	Fold Intensity*^a^*	Expression Type*^b^*	Primer pairs
Solute carrier family 37 (glycerol-3-phosphate transporter), member 2 (SLC37A2)	XM_166184.1	4.23±0.23	B	AGCCTCACCCTGCTCTCCTTCC / CGGGCCTGCTTTTCTGCTTAAT
Ankyrin repeat and SOCS box-containing 2 (ASB2)	NM_016150.3	4.12±0.23	A	CGCCCAGAGTGGACAGTTGGAG / GGCACCCTGTGACAGCAGAAAC
Prosaposin (variant Gaucher disease and variant metachromatic leukodystrophy) (PSAP)	NM_002778.1	4.05±0.12	C	GAATGTGAAGACGGCGTCCGACT / CTCCTCAGTGGCATTGTCCTTCAG
Similar to cytokeratin (AA 1-513) (LOC144501)	XM_096612.7	3.90±0.19	B	AAAGCAGCTTGGAGCCTCTAGGT / GATACTGATATGGAGCGGAGTGGC
Tenascin C (hexabrachion) (TNC)	NM_002160.1	3.68±1.41	A	CATGACTCAGCTGTTGGCAGGT / AAACGTGGTTAAACACCACTGGC
Transmembrane protease, serine 4 (TMPRSS4)	NM_019894.1	3.42±0.29	C	ATTGACAGCACACGGTGCAATG / CCACATGCCACTGGTCAGATTGG
Kallikrein 6 (neurosin, zyme) (KLK6)	NM_002774.2	3.29±0.04	A	GAGGGACCTACGGGCAGCTGTT / CCATGCACCAACTTATTCTGCTCC
Sphingosine kinase 1 (SPHK1)	NM_021972.2	3.03±0.32	A	AGGAAGAGTGGGTTCCAAGACACC / GAGTGCAGCAGTGCCAGGACTAGC
Carbonyl reductase 3 (CBR3)	NM_001236.2	2.85±0.85	C	AACATGTGCAACGAGTTACTGCCG / CCACCAGGTCTCCTTCTGTGAGTG
Hypothetical protein FLJ25348 (FLJ25348)	NM_144569.2	2.78±0.13	B	TCAAGTCTGTTGCTGGCTGTGCT / CATTCGGCCTCCTGTCATCTGG
Midkine (neurite growth-promoting factor 2) (MDK)	NM_002391.2	2.66±0.11	C	AGTGCCTTCTGTCTGCTCGTTAGC / GGCTCTGGGACTCACATTGCTTT
Matrix metalloproteinase 12 (macrophage elastase) (MMP12)	NM_002426.1	2.57±0.14	A	GGGCCCGTATGGAGGAAACATTAT / CCATGCCTGTGTTAATCTTGCTGA
Chromosome 20 open reading frame 108 (C20orf108)	NM_080821.1	2.46±0.66	C	CTGCCCTGTCACGTAGTTGAAGG / CCAGGATGGCCAGTTAATGGTTT
LOC158819 (LOC158819)	XM_098995.1	2.42±0.04	A	TGCCATCTTCTCAGTCCTTCTCC / GACCCAGTAGT AGGCACTGACGGT
**Down-Regulated Genes**				
Homo sapiens heparin-binding growth factor binding protein (HBP17),	NM_005130.1	0.08±0.01	NA	AGCCTCACCCTGCTCTCCTTCC / CGGGCCTGCTTTTCTGCTTAAT
Troponin T3, skeletal, fast (TNNT3)	NM_006757.1	0.13±0.05	NA	AAGGCCAAGGAGCTCTGGGAGA / CTGTGCTTCTGGGCCTGGTCAAT
Dickkopf homolog 1 (Xenopus laevis) (DKK1)	NM_012242.1	0.22±0.001	NA	CCGAGGAGAAATTGAGGAAACCAT / GGCACAGTCTGATGACCGGAGA
ATPase, Na+/K+ transporting, beta 1 polypeptide (ATP1B1)	NM_001677.1	0.22±0.03	NA	TCTTGCCTTGTCCTCCGGTATGT / CAGCACCAGCATGTGATGCCTC
Keratin 6C (KRT6C)	NM_058242.1	0.25±0.03	NA	AGCGTGAGCAGATCAAGACCCTC / ACAACGGCTCCAGGTTCTGCCT
Keratin 5 (epidermolysis bullosa simplex, Dowling-Meara/Kobner/Weber-Cockayne types) (KRT5)	NM_000424.2	0.30±0.07	NA	GAGGCCAAGGTTGATGCACTGAT / CGATGATGCTATCCAGGTCCAGG
CD44 antigen (homing function and Indian blood group system) (CD44)	NM_000610.2	0.34±0.02	NA	GGCTGATCATCTTGGCATCCCT / TTGAGTCCACTTGGCTTTCTGTCC
Dehydrogenase/reductase (SDR family) member 2 (DHRS2)	NM_005794.1	0.36±0.14	NA	TAGAACACTGGCATTGGAGCTGGC / CCCAATCCTCTGCAGCTGATGA
Immediate early response 5 (IER5)	NM_016545.2	0.37±0.03	NA	ACTTTACACCTACCCCTCACCGGA / TCTTCATTGGGAGAGAAAGGTGGG
Serum/glucocorticoid regulated kinase (SGK)	NM_005627.2	0.39±0.01	NA	ATTATGTCGGAGCGGAATGTTCTG / AGCGTTCCCTCTGGAGATGGTAGA
Homo sapiens serpin peptidase inhibitor, clade B (ovalbumin), member 5(SERPINB5)	NM_002639	0.39±0.07	NA	TGAACGACCAGACCAAAATCCTTG / CGTGGCCTCCATGTTCATCATCT
Angiomotin like 2 (AMOTL2)	XM_300852.1	0.40±0.10	NA	AGAAGGATGCAGTGATCAAGGTCC / TCACTAGAAGAGAGCCCTTGCCA
Matrix metalloproteinase 1 (interstitial collagenase) (MMP1)	NM_002421.2	0.42±0.01	NA	GATGGACCTGGAGGAAATCTTGCT / CAAGAGAATGGCCGAGTTCATGA
Plakophilin 1 (ectodermal dysplasia/skin fragility syndrome) (PKP1)	NM_000299.1	0.43±0.05	NA	TTACAGCACCTGCAGTGGTCAGAA / GCAGATCTTGGAGCTGGCCCTAG
Caveolin 1, caveolae protein, 22kDa (CAV1)	NM_001753.3	0.47±0.04	NA	CCCTTAAAGCACAGCCCAGGGA / GGCCTTGTTGTTGGGCTTGTAGA
ATPase, Na+/K+ transporting, beta 3 polypeptide (ATP1B3)	NM_001679.1	0.49±0.17	NA	AGCCTGAAGGAGTGCCAAGGATA / GCAACCAATGGCTGTAGATACCCA
Keratin 19 (KRT19)	NM_002276.2	0.51±0.07	NA	GCCAGGTCAGTGTGGAGGTGGAT / TCCCGGTTCAATTCTTCAGTCCG

a, Fold intensity was obtained from microarray analysis.

b, See the results in Fig. (**2** and **3**). NA, not appricable.

**Table 2.** Clinicopathological Features of 27 OSCC Patients

	Characteristic	No. of Patients
Total		27
Median age (Years)		69(40-90)
Gender	Male	15
	Female	12
Site	Tongue	9
	Gingiva	8
	Buccal mucosa	3
	Floor of mouth	4
	Palate	2
	Sinus	1
T-grades	T1	3
	T2	8
	T3	10
	T4	6
Metastasis		
	Negative	20
	Positive (lymp. N., Post ope)	5
	(Neck )	2
Mode of invasion^#^	YK-1	3
	YK-2	2
	YK-3	11
	YK-4C	7
	YK-4D	0
	ND	4

# Yamamoto-Kohama´s mode of invasion; see referrence [27].

ND, not determined.

**Table 3.** Clinicopathological Features of 64 OSCC Patients

	Characteristic	No. of Patients
Total		64
Median age (Years)		71 (31-91)
Gender	Male	37
	Female	27
Site	Tongue	29
	Gingiva	20
	Buccal mucosa	4
	Floor of mouth	5
	Palate	2
	Sinus	1
	lip	2
	Pharynx	1
T-grades	T1	11
	T2	23
	T3	18
	T4	12
Metastasis		
	Negative	43
	Positive (lymp. N., Post ope)	17
	(Neck )	4
Mode of invasion^#^	YK-1	6
	YK-2	12
	YK-3	27
	YK-4C	14
	YK-4D	5

# Yamamoto-Kohama´s mode of invasion; see referrence [27]

**Table 4.** Correlation of Clinicopathologic Factors and CBR3 Expression in OSCC Tissues. (n = 64)

Factors	CBR3 mRNA^#^		Odds Ratio	95% CI	p-Value^$^
	Low	High			
Age					
<60	10	9			
>60	22	23	1.16	0.40-3.40	<0.79
Gender					
M	14	19			
F	18	13	0.53	0.20-1.43	<0.22
T classification					
T_1-2_	13	19			
T_3-4_	19	13	0.47	0.17-1.27	<0.14
Mode of invasion					
YK1-2	5	14			
YK3-4D	27	19	0.25	0.077-0.82	<0.018^*^
Metastasis					
Node-	21	22			
Node+	11	10	0.87	0.29-2.52	<0.80

# , Low and high means below and above the median value, respectively.

$ , Statistical analysis was performed using the Mann Whitney´s U-test
